# Exploring the Influence of pH on the Dynamics of Acetone–Butanol–Ethanol Fermentation

**DOI:** 10.3390/microorganisms11061610

**Published:** 2023-06-18

**Authors:** Manish Kumar, Supreet Saini, Kalyan Gayen

**Affiliations:** 1 Department of Chemical Engineering, Indian Institute of Technology Gandhinagar, Palaj, Gandhinagar 382055, Gujarat, India; 2 Department of Chemical Engineering, Indian Institute of Technology Bombay, Powai, Mumbai 400076, Maharashtra, India; saini@che.iitb.ac.in; 3 Department of Chemical Engineering, National Institute of Technology Agartala, Agartala 799053, Tripura, India; kalyan.chemical@nita.ac.in

**Keywords:** ABE fermentation, *Clostridium acetobutylicum*, phenomenological model, butanol, acidogenesis, solventogenesis, pH

## Abstract

*Clostridium acetobutylicum* is an anaerobic bacterium that is extensively studied for its ability to produce butanol. Over the past two decades, various genetic and metabolic engineering approaches have been used to investigate the physiology and regulation system of the biphasic metabolic pathway in this organism. However, there has been a relatively limited amount of research focused on the fermentation dynamics of *C. acetobutylicum*. In this study, we developed a pH-based phenomenological model to predict the fermentative production of butanol from glucose using *C. acetobutylicum* in a batch system. The model describes the relationship between the dynamics of growth and the production of desired metabolites and the extracellular pH of the media. Our model was found to be successful in predicting the fermentation dynamics of *C. acetobutylicum*, and the simulations were validated using experimental fermentation data. Furthermore, the proposed model has the potential to be extended to represent the dynamics of butanol production in other fermentation systems, such as fed-batch or continuous fermentation using single and multi-sugars.

## 1. Introduction

*Clostridium acetobutylicum* is a versatile microorganism capable of fermenting both hexose and pentose sugars into biofuels such as butanol and ethanol, as well as other valuable solvents such as acetone, and acids including acetic and butyric acid, and even hydrogen [1]. As a result of this unique ability, researchers have focused on enhancing its potential for producing target chemicals on a global scale [2,3,4]. One of the most recent areas of investigation has been to identify the functional modules of genes involved in the production of acids and solvents, granulose formation, sporulation, sugar uptake systems, and phase transitions, while uncovering their regulatory mechanisms [5]. However, despite significant progress, several challenges remain to be addressed in order to bridge the information gap at both the system and reactor-levels and improve the yield of butanol [6,7,8]. 

On the basis of available physiological and genomic-level information, research efforts must be made towards developing the system and reactor-level understanding of acetone–butanol–ethanol (ABE) fermentation with the help of mathematical models and their validation with experimental data. Precise system-level modeling and simulation can incorporate a large volume of experimental data, and also lead to design of physiologically significant experiments [9]. In this direction, limited data are available to capture the dynamics of fermentation in *C. acetobutylicum* [10].

To advance our understanding of acetone–butanol–ethanol (ABE) fermentation, research efforts should focus on developing mathematical models based on physiological and genomic-level data and validating these models with experimental results. With precise system-level modeling, we can integrate a large volume of experimental data, leading to the design of more physiologically meaningful experiments [9]. By combining mathematical models with empirical data, we can gain a deeper understanding of the ABE fermentation process at both the system and reactor levels. This can help us optimize fermentation conditions and improve product yields. Ultimately, the use of rigorous modeling techniques can enhance our ability to harness the usefulness of ABE fermentation for a range of industrial applications [9,10].

In particular, kinetic studies of ABE fermentation have shown promise in optimizing operational parameters and improving the design of bioreactors for achieving high-yield fermentation processes [11,12,13,14]. To this end, several kinetic models have been constructed for this fermentation process, capturing sugar and production profiles for batch [15,16,17,18], continuous [19,20], and integrated systems with recovery processes [21,22,23]. Some recent efforts have also incorporated detailed biochemical reactions in the metabolic network of the bacterium to enhance modeling accuracy [24,25,26].

ABE fermentation is a complex process influenced by various factors that significantly impact its efficiency and yield. One crucial factor is the composition of the fermentation medium, including the types and concentrations of substrates and nutrients available for the microorganisms. The pH level of the medium also plays a critical role, as it affects the metabolic activities of the microorganisms and the production of desired products. Additionally, the temperature and oxygen availability can significantly influence ABE fermentation, as they influence the growth and metabolic rates of the microorganisms. Other factors such as inoculum cocentration fermentation time, and agitation rate also contribute to the overall fermentation performance [27]. Understanding and optimizing these factors is essential for achieving high ABE production and improving the efficiency of this fermentation process. In this study, we focused on effect of pH level of the medium on *C. acetobutylicum*’s growth and production profiles.

Although media pH is known to be a crucial factor for regulating biomass growth and the transition from acidogenesis to solventogenesis in *Clostridium* cultures [16,28,29], only a limited number of reports on ABE fermentation have investigated this variable. Incorporating pH into a physiological model can provide valuable insights for designing a fermentation process. Some studies have included pH dependence to develop mathematical models that were validated using experimental results from a continuous system [30,31]. However, these studies employed controlled pH profiles that were specific to each phase of fermentation (e.g., 5.7 for acidogenesis and 4.4 for solventogenesis). 

In this study, we propose a phenomenological model to gain insights into the dynamics of ABE fermentation process across different pH profiles. Our model specifically describes the dynamics of biomass growth and synthesis of desired products in a batch process. By accounting for the biphasic metabolic network and variation in pH profiles, our model successfully captures the complexity of the fermentation process. We validate our model through simulations that closely match experimental fermentation data. Moreover, our presented model has the potential to be extended for the analysis of ABE fermentation on consumption of multiple pentose and hexose sugars in other fermentation processes, including fed-batch and continuous systems, both at controlled and uncontrolled pH. The versatility of our model opens up avenues for better understanding the dynamics of fermentation processes, which could lead to more efficient and sustainable industrial processes.

## 2. Materials and Methods 

### 2.1. Model Development

Assumptions for phenomenological modeling of ABE fermentation in *C. acetobutylicum.*

It was assumed that in the absence of any external stress, there is no production of acids in solventogenesis phase and no production of solvents in acidogenesis [32]. By external stress, we mean any external addition of acid/base to control pH.

Acidogenesis shifts to solventogenesis when bacterial growth enters in stationary phase [33]. 

pH variation in culture depends on capacity of buffer (KH_2_PO_4_ and K_2_HPO_4_) and assimilation of produced acids (acetic and butyric acid) during fermentation. 

Effect of buffer (KH_2_PO_4_ and K_2_HPO_4_) was taken in account for maintaining the pH value until phosphate of buffering agents was not consumed by the organism [34,35]. Once the effect of the buffer was exhausted, pH was altered according to the variation in hydrogen ion concentration dissociated from acetic and butyric acid.

High concentration of substrates inhibits synthesis of biomass and butyric acid [25,36,37]. Substrate inhibition was not incorporated in the case of acetic acid as rapid production of acid was observed in the early hours of fermentation experiments while substrate concentration was high.

Various studies report product inhibition in ABE fermentation by acids (acetic and butyric acid) and solvents (acetone, butanol, and ethanol) [37,38]. Therefore, product inhibition was taken in account for modeling production profiles of both metabolic phases in *C. acetobutylicum*. 

Bacterial culture of *C. acetobutylicum* became metabolically inactive once pH fell below 3.0. Metabolically inactivation is meant complete cessation of growth and synthesis of metabolites and substrate utilization. This fact was observed from batch fermentation experiments at uncontrolled pH levels during this study.

### 2.2. pH

It was observed from fermentation experiments that pH of culture remained relatively unchanged for approximately the first 10 h of incubation as assimilation of acids in media starts thereafter. Therefore, pH was unchanged in the starting hours of fermentation because of the buffering effect of buffer agents (KH_2_PO_4_ and K_2_HPO_4_) present in media [35]. Once accumulation of H^+^ ions form produced acids which crossed the buffering zone due to simultaneous production of acetic and butyric acid; *pH* decreased rapidly. In our model, *pH* of buffering zone in early hours of fermentation was determined by Henderson–Hasselbalch’s equation (Equation (1)), while out of the buffering zone it was calculated on the basis of *H^+^* ions dissociated from acids produced (Equation (2)).

(1) $$pH=pKa+log_{10}\left(\frac{Salt-H^{+}}{Acid+H^{+}}\right)$$

(2) $$pH=-log_{10}H^{+}$$

where, $H^{+}=H_{A}^{+}+H_{B}^{+}$. $H_{A}^{+}\;$and$\;H_{B}^{+}$ indicate *H^+^* ions from acetic and butyric acid, respectively. In this biological system, KH_2_PO_4_ and K_2_HPO_4_ act as an acid and a salt, respectively, to generate a buffering zone in media. $H_{A}^{+}$ and $\;H_{B}^{+}$ were calculated as follows:

(3) $$K_{a}^{A}=\frac{\left(H_{int}^{+}+H_{A}^{+}\right)\left(A^{-}\right)}{A_{mol}}$$

(4) $$K_{a}^{B}=\frac{\left(H_{int}^{+}+H_{B}^{+}\right)\left(B^{-}\right)}{B_{mol}}$$

where, $K_{a}^{A}$ and $K_{a}^{B}$ denote dissociation constants for acetic and butyric acid, respectively. $H_{int}^{+}\;$indicates the initial hydrogen ions in the media. Concentrations of acetic acid ($A_{mol}$) with corresponding anion ($A^{-}$) and butyric acid ($B_{mol}$) with related anion ($B^{-}$) played a major role for variation in pH profiles. In our simulations, we considered the initial pH as an input parameter. Subsequently, changes in pH were modeled to account for the presence of buffer agents and acid production during the fermentation process. This approach allowed us to capture the dynamic nature of pH variations throughout the fermentation. By incorporating the influence of buffer agents and acid production, we aimed to provide a more realistic representation of the pH dynamics during ABE fermentation.

### 2.3. pH Effect on Growth and Production Profiles

Activity of enzymes depend on hydrogen ions present in solution and hence, each enzyme acts optimally at a specific value of pH [39].

$$Enzyme.H+S\;K\Leftrightarrow Enzyme.H.S\Rightarrow Enzyme.H+P\;\;Enzyme.H+H^{+}\;\delta^{pH1}\Leftrightarrow Enzyme.H_{2}^{+}\;\;Enzyme.H\;\delta^{pH2}\Leftrightarrow Enzyme^{-}+H^{+}$$

where, S and P indicate substrate and product.

The fact was incorporated in the proposed model and following expression of substrate saturation constant was found successful to confine pH-dependency in all rates involved in the model:

(5) $$K_{j}^{i.pH}=K_{j}^{i}\left(1+\frac{H^{+}}{\delta_{j}^{i.pH1}}+\frac{\delta_{j}^{i.pH2}}{H^{+}}\right)$$

where, $K_{j}^{i}$ and $K_{j}^{i.pH}$ are substrate saturation constant and pH-dependent substrate saturation constant, respectively, for all rates involved in model. i denotes substrate utilized for the production of metabolite j. $H^{+}$ indicates hydrogen ion concentration present in the culture, which was calculated according to the method described in the previous section. $\delta_{j}^{i.pH1}$ and $\;\delta_{j}^{i.pH2}$ denote equilibrium constants for key enzyme(s) when its active sites act as a base and an acid.

### 2.4. Growth

**Figure 1:**
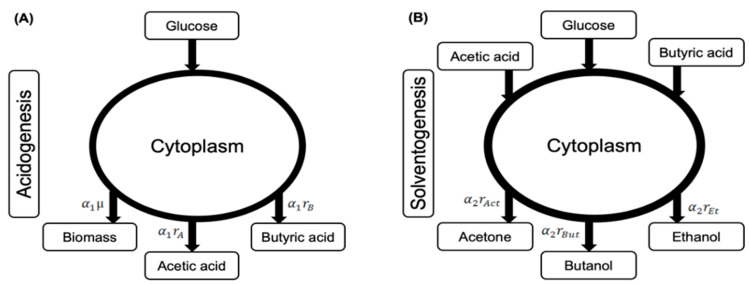
Schematic representation of involvement of substrates and products in both metabolic phases (acidogenesis and solventogenesis) of fermentation in *C. acetobutylicum*. (**A**) Acidogenesis contains one substrate (glucose) and three products (biomass, acetic acid, and butyric acid). (**B**) Solventogenesis phase incorporates three substrates (glucose, acetic acid, and butyric acid) and three products (acetone, butanol, and ethanol). Apart from biomass and metabolites, gases, viz. hydrogen and carbon dioxide are also evolved throughout the fermentation (not shown in figure). During model development, all intracellular steps of metabolic pathway were assumed in steady-state and only extracellular substrates and products were taken into account. The figure also shows rates of production regulated by control coefficients for acidogenesis ($\alpha_{1}$) and solventogenesis ($\alpha_{2}$). To simplify the complex metabolism of *C. acetobutylicum*, we have created a schematic representation that illustrates the two metabolic phases. In order to enhance clarity and readability, we have divided this representation into two separate figures.

*C. acetobutylicum* contains a biphasic metabolic pathway [40]. The first phase of metabolism (acidogenesis) incorporates the exponential growth of the biomass associated with the production of acetic and butyric acid consuming glucose as the sole carbon source. Production of acetone, ethanol, and butanol occurs along with the consumption of glucose, acetic acid, and butyric acid while cells reach the stationary phase. This phase of organism’s metabolism is called solventogenesis [33] (Figure 1).

Biomass growth kinetics can be described in the following equations:

(6) $$\frac{dX}{dt}=\alpha_{1}\left(\mu -K_{d}\right)X$$

(7) $$µ=\frac{{µ}^{max}G}{K_{Biomass}^{G.pH}+G+\frac{G^{2}}{K_{IG}^{X}}}\left(1-\frac{X}{X^{max}}\right)$$

where, X indicates dry biomass (g·L^−1^), and $K_{d}$ is endogenous metabolism coefficient (g·L^−1^), which incorporates the consumption of cell substances (The term $K_{d}X$ is called as cell death rate.). $\alpha_{1\;}$is represented as a control coefficient to regulate the first phase of metabolic pathway (acidogenesis). µ and ${µ}^{max}$ are specific growth rate (h^−1^) and maximum specific growth rate (h^−1^), respectively. G indicates glucose concentration (g·L^−1^). $X^{max}$ represents maximum dry biomass (g·L^−1^) and term $\left(1-\frac{X}{X^{max}}\right)$ describes the decrease in growth due to synthesis of toxic chemicals and depletion of nutrients in the media with the course of fermentation time [11,17]. $K_{Biomass}^{G.pH}$ and$\;K_{IG}^{X}$ are the pH-dependent glucose saturation constant (g·L^−1^) and the substrate inhibition constant for glucose (g·L^−1^), respectively. 

### 2.5. Acidogenesis

Acidogenesis is the first phase of fermentation in *C. acetobutylicum*, which, on consumption of carbon source in the culture media, incorporates rapid biomass growth and synthesis of acids (acetic and butyric acid). Rates of production of acids are described as follows:

(8) $$r_{A}=\frac{r_{A}^{max}G}{(K_{A}^{G.pH}+G)\left(1/\left(1-\frac{A}{K_{IA}}\right)\right)}\;\;\;\;$$

(9) $$r_{B}=\frac{r_{B}^{max}G}{(K_{B}^{G.pH}+G+\frac{G^{2}}{K_{IG}^{B}})\left(1/\left(1-\frac{B}{K_{IB}}\right)\right)}$$

where, $r_{A}$ and $r_{B}$ are rate of synthesis for acetic (A) and butyric (B) acid (h^−1^), respectively. $r_{A}^{max}$ and $r_{B}^{max}$ are maximum rates of synthesis for acetic and butyric acid (h^−1^) and their pH-dependent saturation constants (g·L^−1^) are indicated as $K_{A}^{G.pH}$ and $K_{B}^{G.pH},\;$respectively. Previous reports postulated that accumulation of acids is one of the critical factors which create cellular stress resulting in metabolic transition to solventogenesis [41]. Additionally, acids control their own production via negative feedback loops [32]. To account for this fact in rates of acid production, two product inhibition constants, (g·L^−1^) as $K_{IA}$ and $K_{IB}$, are introduced for acetic and butyric acid, respectively. Previous results from fermentation experiments illustrated that butyric acid was produced at a lower rate than acetic acid and its expression only took place after a lag in the early hours of fermentation. For capturing these features of the fermentation dynamics, substrate inhibition was taken in account in the rate of butyric acid production (unlike in the rate of acetic acid formation).$\;K_{IG}^{B}$ denotes glucose inhibition constant (g·L^−1^) for butyric acid production.

### 2.6. Solventogenesis

In the solventogenesis phase, the organism consumes the produced acids in the acidogenesis phase along with the carbon source in the media and produces solvents (acetone, butanol, and ethanol) (Figure 1). It was observed from fermentation experiment that consumption of glucose and both acids occurred simultaneously. The fact was included in following rate equation of solvent production:

(10) $$r_{i}=\frac{r_{i}^{G.max}r_{i}^{A.max}r_{i}^{B.max}GAB}{\left(G+K_{i}^{G.pH}\right)\left(A+K_{i}^{A.pH}\right)\left(B+K_{i}^{B.pH}\right)\left(1/\left(1-\frac{i}{K_{Ii}}\right)\right)}$$

where, i stands for acetone, butanol, and ethanol. $r_{i}^{G.max}$, $r_{i}^{A.max}$, and $r_{i}^{B.max}$ denote maximum rate of solvent production(h^−1^) for glucose, acetic acid, and butyric acid, respectively. $K_{i}^{G.pH}$,$\;K_{i}^{A.pH}$, and $K_{i}^{B.pH}$ are pH-dependent glucose, acetic acid, and butyric acid saturation constants for solvents production (g·L^−1^). $K_{Ii}$ symbolizes product inhibition constant for solvents. Equation (10) was found successful to incorporate the simultaneous consumption of three substrates [42]. 

Apart from acids and solvents, hydrogen and carbon dioxide are also the evolved in both phases of ABE fermentation. Hydrogenase catalyzes oxidation of reduced ferredoxin for hydrogen production. Carbon dioxide is evolved through two conversions, pyruvate to acetyl-CoA and acetoacetate to acetone catalyzed by pyruvate-ferredoxinoxidoreductase and acetoacetate decarboxylase, respectively [43]. Consumption of glucose in acidogenesis and simultaneous consumption of glucose, acetic acid, and butyric acid in solventogenesis phase yield carbon dioxide. In addition, hydrogen is also released throughout the fermentation with lower rate than carbon dioxide [41]. Rates of hydrogen and carbon dioxide evolution in both phases are described as follows:

(11) $$r_{H_{2}}^{G}=\frac{r_{H_{2}}^{G.max}G}{K_{H_{2}}^{G.pH}+G}$$

(12) $$r_{CO_{2}}^{G}=\frac{r_{CO_{2}}^{G.max}G}{K_{CO_{2}}^{G.pH}+G}$$

(13) $$r_{CO_{2}}^{AB}=\frac{r_{CO_{2}}^{A.max}r_{CO_{2}}^{B.max}AB}{\left(A+K_{CO_{2}}^{A.pH}\right)\left(B+K_{CO_{2}}^{B.pH}\right)}$$

where, rate and maximum rate of hydrogen evolution are denoted as $r_{H_{2}}^{G}$ and $r_{H_{2}}^{G.max},$ respectively, and $K_{H_{2}}^{G.pH}$ is glucose saturation constant for hydrogen evolution. $r_{CO_{2}}^{G}$ and $r_{CO_{2}}^{AB}$ represent the rate of carbon dioxide evolution (h^−1^) from glucose alone and from simultaneous utilization of acetic acid + butyric acid, respectively. $r_{CO_{2}}^{G.max}$, $r_{CO_{2}}^{A.max}$, and $r_{CO_{2}}^{B.max}$ denote maximum carbon dioxide evolution rate (h^−1^) from glucose, acetic acid, and butyric acid, respectively. $K_{CO_{2}}^{G.pH}$, $K_{CO_{2}}^{A.pH}$, and $K_{CO_{2}}^{B.pH}$ are pH-dependent glucose, acetic acid, and butyric acid saturation constants (g·L^−1^) for carbon dioxide evolution.

### 2.7. Ordinary Differential Equations Encompassing Mass Balance of Fermentation

Following equations were constructed to represent the growth and production profiles of fermentation:

#### 2.7.1. Consumption of Glucose

Glucose was used as a main carbon source in this fermentation for yielding biomass and other metabolites. More precisely, glucose is the sole carbon source in acidogenesis, while glucose is utilized simultaneously with acetic and butyric acid for synthesizing solvents in solventogenesis. Therefore, the consumption rate of glucose for production of biomass, acids, solvents, and carbon dioxide is represented as follows:

(14) $$-\frac{dG}{dt}=-Y_{\frac{G}{X}}\alpha_{1}µ\;X-Y_{\frac{G}{A}}\alpha_{1}r_{A}\;X-Y_{\frac{G}{B}}\alpha_{1}r_{B}\;X-Y_{\frac{G}{Act}}\alpha_{2}r_{Act}\;X-Y_{\frac{G}{Et}}\alpha_{2}r_{Et}\;X\;-Y_{\frac{G}{But}}\alpha_{2}r_{But}\;X-Y_{\frac{G}{CO_{2}}}r_{CO_{2}}^{G}\;X$$

where, $Y_{\frac{G}{X}}$, $Y_{\frac{G}{A}}$,$\;Y_{\frac{G}{B}}$, $Y_{\frac{G}{Act}}$, $Y_{\frac{G}{Et}}$, $Y_{\frac{G}{But}}$, and $Y_{\frac{G}{CO_{2}}}$ denote the yield coefficients (g·g^−1^) of glucose consumption with respect to biomass, acetic acid, butyric acid, acetone, ethanol, butanol formation, and carbon dioxide evolution, respectively. $\alpha_{1}$ and $\alpha_{2}$ indicate control coefficients for acidogenesis and solventogenesis, respectively. 

#### 2.7.2. Synthesis and Consumption of Acetic and Butyric Acid

*C. acetobutylicum* produces acetic and butyric acid utilizing glucose as the sole carbon source in the acidogenesis phase of metabolism, and thereafter consumes the produced acids simultaneously with glucose in second phase for solvent production. Mole balances of acetic and butyric acid are represented as follows in ordinary differential equations:

(15) $$\frac{dA}{dt}=\alpha_{1}r_{A}X-Y_{\frac{A}{Act}}\alpha_{2}r_{Act}\;X-Y_{\frac{A}{Et}}\alpha_{2}r_{Et}\;X-Y_{\frac{A}{But}}\alpha_{2}r_{But}\;X-Y_{\frac{A}{CO_{2}}}\alpha_{2}r_{CO_{2}}^{AB}\;X$$

(16) $$\frac{dB}{dt}=\alpha_{1}r_{B}X-Y_{\frac{B}{Act}}\alpha_{2}r_{Act}\;X-Y_{\frac{B}{Et}}\alpha_{2}r_{Et}\;X-Y_{\frac{B}{But}}\alpha_{2}r_{But}\;X-Y_{\frac{B}{CO_{2}}}\alpha_{2}r_{CO_{2}}^{AB}\;X\;\;$$

where, $Y_{\frac{A}{Act}}$, $Y_{\frac{A}{Et}}$, $Y_{\frac{A}{But}}$, and $Y_{\frac{A}{CO_{2}}}$ indicate the yield coefficients (g·g ^−1^) of acetic acid consumption with respect to acetone, ethanol, butanol formation, and carbon dioxide evolution. Similarly, $Y_{\frac{B}{Act}}$, $Y_{\frac{B}{Et}}$, $Y_{\frac{B}{But}}$, and $Y_{\frac{B}{CO_{2}}}$ indicate the yield coefficients (g·g ^−1^) of butyric acid consumption with respect to acetone, ethanol, butanol formation, and carbon dioxide evolution. 

#### 2.7.3. Synthesis of Acetone, Ethanol, Butanol

The bacterium grows rapidly in acidogenesis and produces acids. Accumulation of acids drives a significant decrease in pH in media leading causes toxic effect for cells. To overcome the toxic effect from produced acids, cells shift their metabolism to solventogenesis, which leads to consumption of acids and synthesis of solvents. The production of solvents can be written as the following equations:

(17) $$\frac{d\left(Act\right)}{dt}=\alpha_{2}r_{Act}X\;\;\;\;\;\;\;\;\;\;$$

(18) $$\frac{d\left(Et\right)}{dt}=\alpha_{2}r_{Et}X$$

(19) $$\frac{d\left(But\right)}{dt}=\alpha_{2}r_{But}X\;$$

#### 2.7.4. Hydrogen Evolution and Carbon Dioxide Evolution

*C. acetobutylicum* releases hydrogen and carbon dioxide in both phases of metabolism. It has been reported that hydrogen evolution rate is higher in acidogenesis than solventogenesis and carbon dioxide evolution rate is approximately similar in both phases [41,43]. Following equations represent the evolution of hydrogen and carbon dioxide:

(20) $$\frac{d\left(H_{2}\right)}{dt}=r_{H_{2}}^{G}X$$

(21) $$\frac{d\left(CO_{2}\right)}{dt}=\left(r_{CO_{2}}^{G}+\alpha_{2}r_{CO_{2}}^{AB}\right)X$$

#### 2.7.5. Consumption of Phosphate

Previous studies reported that *KH*_2_*PO*_4_ and *K*_2_*HPO*_4_ act as buffer agents in bacterial media [35]. However, *KH*_2_*PO*_4_ and *K*_2_*HPO*_4_ are also the source of phosphate during metabolic activities of organism [34]. Therefore, we took phosphate consumption and its effect on buffer strength of solution into account for modeling the pH profile of fermentation. Equations (22) and (23) represent consumption of *KH*_2_*PO*_4_ and *K*_2_*HPO*_4_, respectively.

(22) $$\frac{d\left(KH_{2}PO_{4}\right)}{dt}=-\gamma_{KH_{2}PO_{4}}\mu X-m_{KH_{2}PO_{4}}X\;\;\;\;$$

(23) $$\frac{d\left(K_{2}HPO_{4}\right)}{dt}=-\gamma_{K_{2}HPO_{4}}\mu X-m_{K_{2}HPO_{4}}X$$

where, $\gamma_{KH_{2}PO_{4}}$ and $\gamma_{K_{2}HPO_{4}}$ are the control coefficients for consumption of *KH*_2_*PO*_4_ and *K*_2_*HPO*_4_, respectively. $m_{KH_{2}PO_{4}}$ and $\;m_{K_{2}HPO_{4}}$ are maintenance coefficients of *KH*_2_*PO*_4_ and *K*_2_*HPO*_4_ for biomass, respectively.

### 2.8. Experimental Data

The experimental data used in this study for parameter estimation and model validation were obtained from our previously published paper [32]. The reference paper provides detailed descriptions of all experimental procedures. In this study [32], all fermentation experiments were conducted at a temperature of 37 °C in triplicate. To measure the biomass, optical density (A_600_) was utilized as a proxy, which was then converted to dry cell weight (g/L) using a correlation curve established between the absorbance measured at 600 nm and the corresponding dry cell weight. Specifically, Gholizadeh el al. [44] determined that one unit of OD_600_ roughly corresponded to 0.79 g/L of dry cell weight for *C. acetobutylicum* cells.

### 2.9. Validation of Model

**Figure 2:**
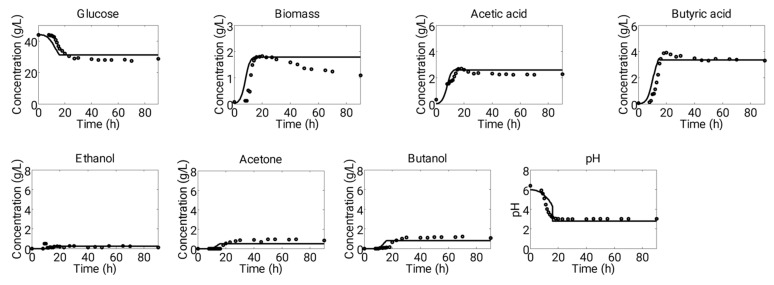
Depiction of a dynamics of 90 h long batch fermentation system using *C. acetobutylicum* at starting pH of 6.4 (“Experiment 1”). Figure shows comparative scenario of experimental (dots) and simulation (solid lines) data of fermentation. This set of experimental data was used to estimate the parameters of proposed model.

**Figure 3:**
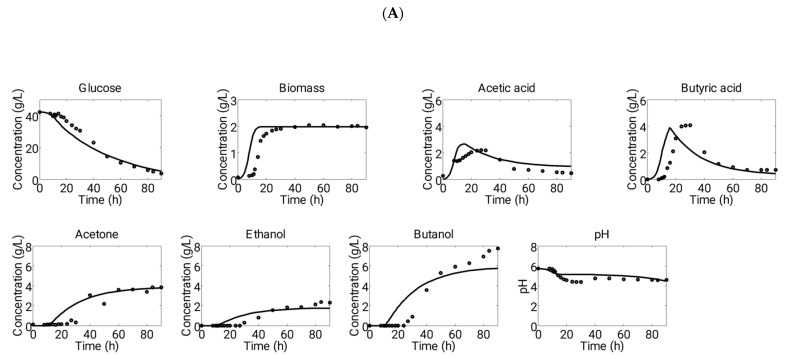

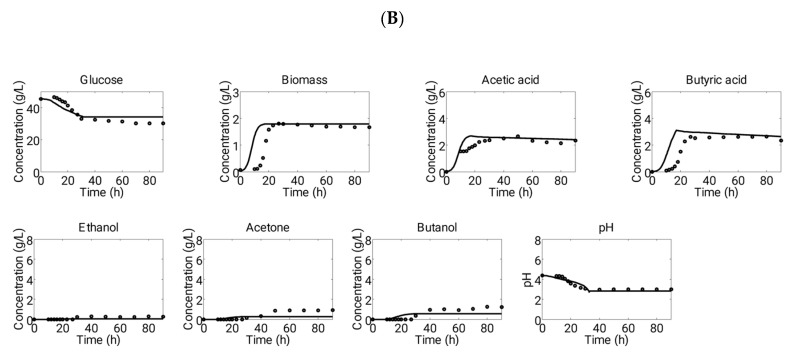
Demonstration of comparative profiles of experimental (dots) and simulation (solid lines) data of ABE fermentation in a batch process at starting of (**A**) pH 5.7 (“Experiment 2”) and (**B**) pH 4.4 (“Experiment 3”). These sets of experimental data were used to validate the predictions of model.

Model parameters were estimated by fitting the data set of Experiment 1, and data of Experiment 2 and Experiment 3 were used to validate the model prediction. Ordinary differential equations (Equations (1)–(25)) were solved by using ODE 15 s solver available in MATLAB (Mathworks, Natick, MA, USA) for simulating experimental results. Figure 2 and Figure 3 show a comparative fit of model predictions to experimental data.

## 3. Results

Numerous reports have suggested that the pH level of the fermentation medium plays a crucial role in regulating the physiological behavior of *Clostridium acetobutylicum* during acetone–butanol–ethanol (ABE) fermentation [31,45]. It has been observed that the optimal pH range for acidogenesis is between 5.0–6.0, while for solventogenesis, it lies in the range of 4.0–5.0 [45]. In light of these findings, we have developed a physiological model that accounts for the pH profile in conjunction with other extracellular metabolites during ABE fermentation. The model, as illustrated in Figure 1, incorporates two control strategies: (a) metabolic transition from acidogenesis to solventogenesis is regulated through biomass growth, with acidogenesis occurring during the exponential phase and solventogenesis during the stationary phase, as supported by experimental data; and (b) the rates of metabolite formation are controlled by the organism’s modulation of its metabolism for optimum growth and synthesis of metabolites in both phases, according to different pH levels in the medium. We have employed three sets of fermentation experimental data, starting with initial pH values of 6.4, 5.7, and 4.4, which are referred to as “Experiment 1,” “Experiment 2,” and “Experiment 3,” respectively, throughout the text of the paper [32].

The pH of a fermentation media was determined by calculating the hydrogen ion concentration dissociated from two acids, acetic acid (Ka = 1.7378 × 10^−5^) and butyric acid (Ka = 1.5136 × 10^−5^). During the initial phase of the fermentation (up to about 10 h), the pH remained stable due to the buffer capacity provided by KH_2_PO_4_ and K_2_HPO_4_ (0.75 g·L^−1^) present in the media. Experimental observations indicated that during this period, a small amount of acetic acid (1.4–1.7 g·L^−1^) and butyric acid (0.08–0.7 g·L^−1^) were produced, and the H^+^ ions produced from the acids were balanced by the buffer agents to maintain a constant pH. However, after this initial phase, the pH of the media rapidly decreased due to the production of acids, and the buffer was no longer effective in controlling the pH (Figure 2 and Figure 3).

**Figure 4:**
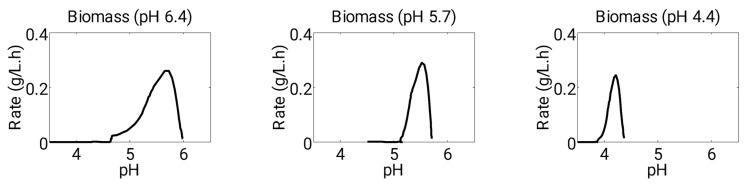
Rates of biomass accounting the variation in starting pH level.

Furthermore, variations in culture performance due to different starting pH values were incorporated into a model using an expression that related the substrate saturation constant and the hydrogen ion concentration present in the culture (Equation (5)). The values of equilibrium constants ($\delta_{j}^{i.pH1}$ and $\delta_{j}^{i.pH2}$) used in the model were determined by optimizing the production rates of individual products at a specific pH (Table 1). The suitability of this expression for accounting for the effect of pH variation in the rates of products was demonstrated through comparative observations between simulation and experimental data (Figure 4). The following sections of the study include the estimation of model parameters, details of the control strategy, and outcomes of model simulations, and their comparison with experimental data.

### 3.1. Estimation of Model Parameters

The model parameters were estimated by fitting the fermentation data of Experiment 1, which had an initial pH of 6.4 (Table 1). Our approach involved an iterative process, where we adjusted the parameter values until the simulation data aligned well with the experimental data. Subsequently, the model, incorporating these estimated parameter values, was utilized to simulate the growth, consumption, and production profiles under different pH conditions. Preliminary attempts were made, and the parameters were improved using a dynamic optimization algorithm called “fmincon” in MATLAB (Mathworks, Natick, MA, USA). The estimated model parameters can be found in Table 1. The initial values of solvents (acetone, butanol, and ethanol) and gases (hydrogen and carbon dioxide) were set to zero, as it was assumed that inoculums in the late exponential phase were used during the experiments before entering the solventogenesis phase. The initial values of all variables are reported in Table 2. The initial values of substrates (glucose, KH_2_PO_4_, and K_2_HPO_4_) were taken from experimental data, while the initial values of all other variables were estimated based on the fitting of experimental data from Experiment 1.

### 3.2. Control Mechanism for Metabolic Switch from Acidogenesis to Solventogenesis

Various fermentations were carried out at different uncontrolled pH, and it was found that acid production only occurred during the exponential phase of biomass growth, while solvent production began when the growth entered the stationary phase. Therefore, the transition of biomass growth from the exponential to the stationary phase was used as an indicator for the metabolic shift in the development of the current model. To this end, two control coefficients ($\alpha_{1}$ and $\alpha_{2}$) were included in the model for the two metabolic phases. $\alpha_{1}$ was used to control acid production, while $\alpha_{2}$ was used to control solvent production. The coefficients were assigned values of 0 and 1 to represent the on and off states for maintaining only one active phase at a given time. Additionally, it was observed that the time for metabolic switching varied from approximately 18 to 30 h in different sets of experimental data and depended on the growth conditions. The following inequality expressions were used in model to confine the metabolic shift:

For acidogenesis

(24) $$\alpha_{1}=1;\;if\;0\leq \frac{X}{X^{max}}\leq R_{A}\;and\;\alpha_{2}=0;\;if\;\frac{X}{X^{max}}>R_{S}\;\;\;\;\;\;$$

**Figure 5:**
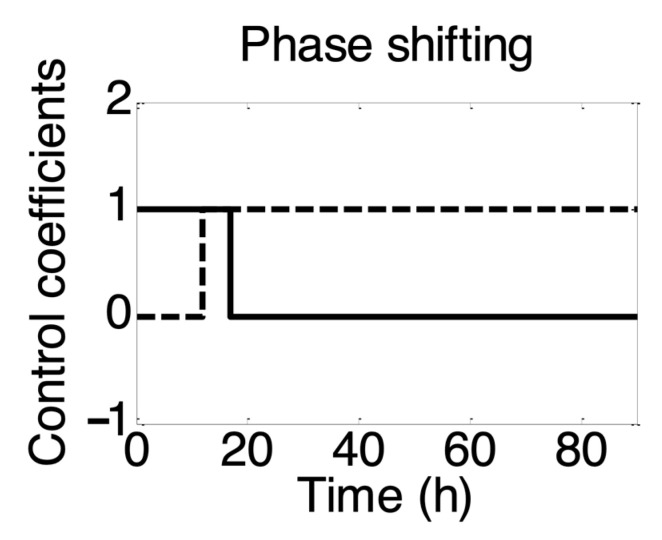
Representation of control strategy used in model to confine the metabolic transition from acidogenesis to solventogenesis. Figure shows profiles of control coefficients for acidogenesis ($\alpha_{1}$, solid lines) and solventogenesis ($\alpha_{2}$, dotted lines) over the time course of fermentation. The values of 0 and 1 of control coefficients ($\alpha_{1}$ and $\alpha_{2}$) embrace switch-off and -on of production of each metabolite from specific substrate(s) consistent with the decision-making mechanism of the organism.

For solventogenesis

(25) $$\alpha_{2}=1;\;if\;0\leq \frac{X}{X^{max}}\leq R_{S}\;and\;\alpha_{1}=0;\;if\;\frac{X}{X^{max}}>R_{A}\;\;\;\;\;$$

where, $R_{A}$ and $R_{S}\;$represent constant ratios of biomass concentration and maximum biomass concentration for acidogenesis and solventogenesis respectively. Their values ($R_{A}=0.99\;$and $\;R_{S}=0.82$) were estimated on the basis on experimental data (from Experiment 1). The inequalities were such that value of both control coefficients was 1 when $\frac{X}{X^{max}}$ lies between $R_{A\;}$and $R_{S}$. This means that both metabolic phases were active in this interval, consistent with experimental observations. Individually, acidogenesis and solventogenesis were active before ($\alpha_{1}$ = 1 and $\alpha_{2}$ = 0) and after ($\alpha_{1}$ = 0 and $\alpha_{2}\;$ = 1) this interval respectively (Figure 5).

### 3.3. Acidogenesis Incorporates Rapid Biomass Growth and Production of Acids over the Consumption of Glucose

The production kinetics of biomass was modeled using Equations (1) and (2). Experimental measurements indicated that maximum biomass concentrations ranging from 1.8–2.0 g·L^−1^ were achieved under different initial pH conditions. The close proximity of the maximum biomass concentrations over a significant change in media pH (4.5–6.8) suggests that growth is relatively insensitive to initial pH variations in this range. However, low biomass growth was observed during the fermentation process, which can be attributed to the presence of toxic chemicals in the media and substrate inhibition. To account for the slow biomass growth, a term of $\left(1-\frac{X}{X^{max}}\right)$ and substrate inhibition were incorporated into the growth kinetics model. The substrate inhibition constant was determined to be 170 g·L^−1^. Acid production rates were simulated using Equations (3) and (4). Experimental data indicated that the rate of acetic acid formation was higher than butyric acid in the early stages of acidogenesis [32]. To account for the delay in butyric acid synthesis, substrate inhibition was included in the model. Additionally, the well-known phenomenon of product inhibition was successfully incorporated into the rate expressions [18].

Figure 2 presents the simulation results in comparison with the fermentation data obtained from Experiment 1. During this experiment, the initial pH of 6.4 was maintained, but due to the production of a considerable amount of acetic acid (2.7 g·L^−1^) and butyric acid (3.9 g·L^−1^) in the first metabolic phase, the pH level dropped to 3.0. The metabolic activity of cells ceased once the pH level went below 3.0. During this phase, the maximum concentration of acids recorded during this phase was 1.8 g·L^−1^, while approximately 10 g·L^−1^ of glucose was consumed. Acid production was halted after 18 h of fermentation time, and at the same time, the biomass entered the stationary phase.

Subsequently, the model was simulated using the inputs obtained from Experiment 2. The comparison between the predictions generated by the model and the measurements can be observed in Figure 3A. The pH was initially set to a value of 5.7 during the simulations and was not controlled thereafter, mimicking the experimental conditions. The production of acid led to a further reduction in pH, reaching a final value of 4.4. It is noteworthy that the cellular metabolism remained active throughout the remaining fermentation duration despite the acidic conditions. The acidogenesis phase resulted in the detection of total concentrations of acetic and butyric acid, amounting to 2.2 and 4.0 g·L^−1^, respectively, while approximately 10 g·L^−1^ of glucose was consumed. Notably, acid production persisted for a longer duration of 27 h compared to Experiment 1.

To mimic the growth conditions described in Experiment 3, we conducted simulations using an initial pH of 4.4 and a glucose concentration of 45.39 g·L^−1^, as detailed in Table 2. The resulting model outcomes were compared to experimental measurements (Figure 3B). During the experiment, the pH decreased from 4.4 to 3.0, negatively impacting cellular metabolic activities. This pH profile facilitated the display of dynamic metabolite profiles by the cells, similar to those observed in Experiment 1. Acid production occurred for up to 30 h, resulting in final concentrations of 2.3 g·L^−1^ 1 and 2.5 g·L^−1^ for acetic acid and butyric acid, respectively. Although acid production continued for the full 30 h, it was lower than in the other two pH profiles, possibly due to the cells already being under stress from the low starting pH. This led to a decreased metabolic rate.

Upon reaching its peak, the biomass elicited a shift in metabolism from acidogenesis to solventogenesis. In terms of modeling, during the second phase of metabolism (solventogenesis), the values of phase control coefficients, $\alpha_{1}$ became 0 and $\alpha_{2}$ was set to 1. In the brief interim period between acidogenesis and solventogenesis, both phase control coefficients were set to 1, indicating the simultaneous occurrence of active acidogenesis and solventogenesis. This approach proved advantageous in delineating the variance in switch-time between different growth conditions, as depicted in Figure 4.

### 3.4. Production of Solvents Is Arisen in Solventogenesis Phase over the Consumption of Glucose and Acids Produced in Acidogenesis Phase

During the solventogenesis phase, cells consume the acids produced in the acidogenesis phase along with the glucose present in the growth medium. In our proposed model, the formation rate of solvents is described by Equation (5), which comprises three distinct maximum rates and three substrate saturation constants for each solvent derived from all three substrates. Based on experimental observations, we determined that glucose, acetic acid, and butyric acid are all utilized simultaneously for solvent production, as illustrated in Figure 3A, and this was incorporated into our kinetic model. Additionally, our rate expression for solvent production takes into account product inhibition.

In Experiment 2, which had an initial pH of 5.7, a greater amount of solvents (acetone, butanol, and ethanol) were produced compared to Experiments 1 and 3. The final concentrations of these solvents were recorded as 3.8 g·L^−1^, 2.3 g·L^−1^, and 7.7 g·L^−1^, respectively. This pH profile allowed for the cells to remain metabolically active throughout the fermentation process and to consume glucose, acetic acid, and butyric acid until they were almost completely exhausted. As a result of this acid consumption, the pH of the medium slightly increased from its lowest value of 4.4 to 4.6 (Figure 3A). In contrast, Experiments 1 (initial pH of 6.4) and 3 (initial pH of 4.4) exhibited very low production of solvents. In Experiment 1, the concentrations of acetone, butanol, and ethanol were 0.96 g·L^−1^, 1.2 g·L^−1^, and 0.25 g·L^−1^, respectively. In Experiment 3, their concentrations were recorded as 0.91 g·L^−1^, 1.23 g·L^−1^, and 0.28 g·L^−1^, respectively. The low solvent production in these fermentations was due to a significant decrease in pH to a level of 3.0, which was unfavorable for metabolic activity in the cells. Collectively, during Experiments 1 and 3, the cells were unable to maintain a favorable pH level for solvent production, whereas in Experiment 2, the cells performed well at an initial pH of 5.7. Therefore, an uncontrolled initial pH of 5.7 was found to be favorable for higher solvent production and for maintaining the cells in an active state for a longer period in a batch system.

### 3.5. Hydrogen and Carbon Dioxide Are Evolved in Both Metabolic Phases

**Figure 6:**
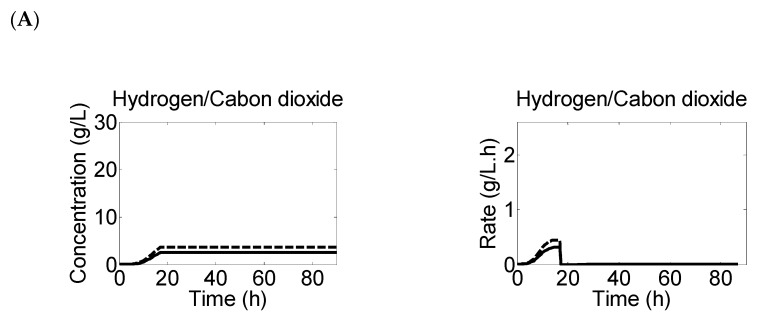

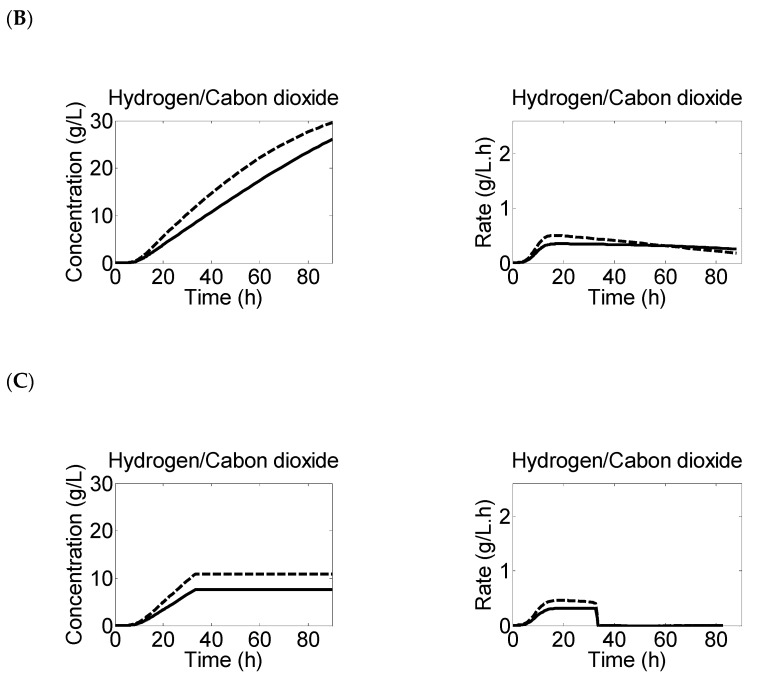
The figure shows predicted data of rates and production profiles of hydrogen (solid lines) and carbon dioxide (dotted lines) evolution at starting pH of (**A**) 6.4 (“Experiment 1”) (**B**) 5.7 (“Experiment 2”) and (**C**) 4.4 (“Experiment 3”).

In this study, we also aimed to predict the dynamics of hydrogen and carbon dioxide evolution across various pH profiles during fermentation experiments. The results are presented in Figure 6, which illustrates the evolution and rate profiles for gases during the experiments. The findings suggest that the evolution of gases stopped in Experiment 1 and 3 when the culture reached a pH of 3.0. The concentration of gases in both experiments remained below 15 g·L^−1^, which was released during acidogenesis and short solventogenesis stages before the metabolic activities ceased due to stressed growth conditions. However, Experiment 2 displayed higher concentrations of hydrogen (26 g·L^−1^) and carbon dioxide (30 g·L^−1^) during both phases of metabolism, as cells were functional up to the exhaustion of available carbon sources.

**Figure 7:**
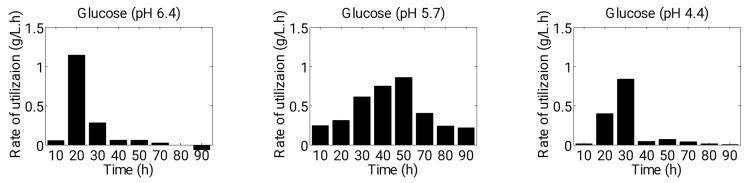
Variation in consumption rate of glucose at different starting pH levels of 6.4, 5.7 and 4.4.

**Figure 8:**
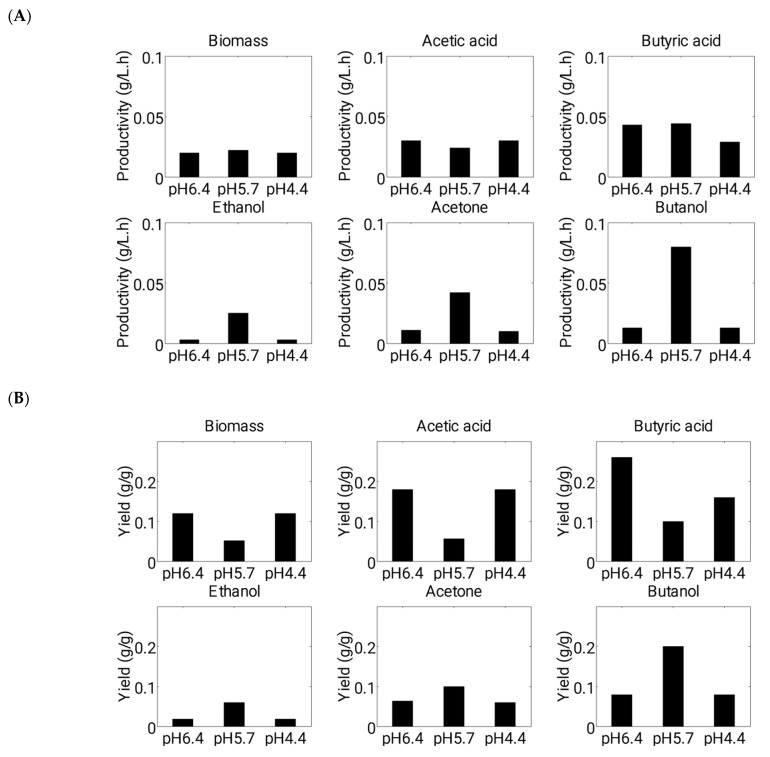
Figure shows (**A**) productivity and (**B**) yield of batch fermentation runs at different initial pH using *C. acetobutylicum*.

Furthermore, we conducted an analysis of glucose consumption rate, as well as the productivity and yield of fermentation products under various growth conditions (Figure 7 and Figure 8A,B). During acidogenesis, the consumption rate of glucose (g·L^−1^·h^−1^) was found to be higher in Experiment 1 and 3 compared to Experiment 2 (up to approximately 30 h) (Figure 7). Specifically, the starting pH of 6.4 enabled cells to consume glucose rapidly in the early stages of fermentation, as cells exhibited a higher growth rate and acid production at this pH level. However, after 30 h (during solventogenesis), the consumption rate of glucose was almost negligible at starting pH levels of 6.4 and 4.4 due to the unfavorable pH level (pH 3.0) reached by the culture. At a starting pH of 5.7, cells consumed a greater amount of glucose during solventogenesis than in acidogenesis, as carbon flow favored solvent production. The highest consumption rate was observed between 40 and 50 h of fermentation time, after which the rates decreased due to the accumulation of toxic products and a reduced amount of remaining glucose in the batch fermenter (Figure 7).

Additionally, our evaluation of yield (g·g^−1^) and productivity (g·L^−1^·h^−1^) revealed that, in Experiments 1 and 3, cells were more involved in acidogenesis than solventogenesis, while the opposite was observed in Experiment 2 (Figure 8A,B). At a starting pH of 5.7, both yield and productivity values were approximately two-fold higher than those observed at starting pH levels of 6.4 and 4.4. Thus, our modeling and experimental approaches suggested that a high starting pH level (6.4) may promote a high rate of biomass and acid production to shorten the metabolic shift time. However, to maintain cells in an active condition and produce a greater amount of solvents in the second phase, an intermediate pH level should be maintained above a certain threshold (pH 4.4).

## 4. Discussion

The model was successful in predicting the profiles of glucose consumption, biomass formation, acids’ and solvents’ production, pH, and gases (hydrogen and carbon dioxide) evolution in a batch fermentation system of *C. acetobutylicum*. The model was used to predict fermentation profiles at starting pH levels of 5.7 and 4.4, and these predictions were validated using fermentation data at both pH levels. Our study emphasizes the crucial role of pH in achieving optimal biomass yield of *C. acetobutylicum* and maximizing solvent production. Additionally, the data predicted by our model suggests, that while a high biomass yield does not necessarily result in high solvent concentrations, rapid growth during the initial hours of a batch fermentation promotes increased acid production (Fig. 8B). However, if the objective is to enhance acetic and butyric acid yield instead of solvent production, it is favorable to prioritize high growth during the early stages of fermentation to yield higher acid yields.

The results showed that a starting pH of 5.7 was more suitable than pH levels of 6.4 and 4.4 for higher production of solvents. In the proposed model, it was found that when the starting pH was higher than the optimum pH (pH 5.7), the cells grew faster and produced acids rapidly, which led to shock and caused the cells to enter sporulation instead of solventogenesis. Similarly, when the cells grew at a lower pH level than the optimum level, a small amount of acids lowered the pH level significantly and forced the cells to enter sporulation. Therefore, due to the initialization of sporulation, cells produced lower concentrations of solvents at higher and lower starting levels of pH than pH 5.7.

It has been observed that sporulation and solventogenesis are regulated by one activator, Spo0A [46]. However, the reasons for Spo0A activating sporulation or solventogenesis under different growth conditions are unknown [47]. At a starting pH level of 5.7, the cells were able to hold the pH at a certain favorable level (pH 4.4) for growth through the consumption of acids. Therefore, in this case, the cells performed metabolically well and consumed glucose and acids up to almost exhaustion for producing high concentrations of solvents. Our study revealed a significant impact of pH level on the utilization rate of glucose (g/L·h) during fermentation. Specifically, we observed that maintaining a pH level of 5.7 facilitated continuous glucose consumption throughout the process, resulting in a higher yield of solvents (Figure 7 and Figure 8B).

The maximum concentration of biomass achieved in the batch fermentation was not found to be very sensitive to the starting pH level as the biomass achieved a similar maximum concentration for all three uncontrolled growth conditions. Additionally, biomass growth showed a decline in the case of a starting pH of 6.4, which might be due to the rapid biomass and acids production rate compared to the other pH levels. However, this decline in biomass growth was not predicted by the proposed model. Along with the profiles of other products, the model was also capable of predicting the concentration of hydrogen and carbon dioxide in both phases of metabolism. Furthermore, it was observed from the predicted profiles that under the starting pH of 5.99, solventogenesis was active for 65 h, which is around five-fold higher than the period for the starting pH of 6.8 and 4.5. While the activation time of acidogenesis was similar in all three different pH environments, it indicates that the activation period of the solventogenesis phase is dependent on the starting pH level.

## 5. Conclusions

We developed an uncontrolled pH-based kinetic model to capture the fermentation dynamics of *C. acetobutylicum* in a batch system. The model accurately described the consumption of glucose, formation of biomass, production of acids and solvents, pH changes, and evolution of hydrogen and carbon dioxide gases under various experimental conditions. Our study revealed that maintaining a pH above 4.4 is crucial for keeping the cells in a metabolically active state, resulting in higher solvent production during the second phase of fermentation. This model can be extended to represent the dynamics of other types of fermentation processes, such as fed-batch and continuous systems, both with controlled and uncontrolled pH. Furthermore, the proposed model can aid in developing the kinetics of ABE fermentation using other sugars, such as arabinose and xylose, either individually or in combination with glucose. While significant efforts are still needed, our study provides a crucial foundation for identifying optimal parameters in the production of biobutanol at an industrial scale. By elucidating the relationship between medium pH and the production of biobutanol, our findings contribute to the development of efficient and scalable processes. Although additional research and optimization are necessary, our study serves as an important steppingstone towards realizing the industrial production of biobutanol.

## Figures and Tables

**Table 1:** Estimated parameter values used in proposed model.

Parameter	Unit	Value	Parameter	Unit	Value	Parameter	Unit	Value
${µ}^{max}$	h^−1^	0.78	$K_{Act}^{G}$	g·L^−1^	3.2	$Y_{\frac{G}{Act}}$	g·gg·g^−1^	1.07
$K_{IG}^{X}$	g·L^−1^	170	$\delta_{Act}^{S.pH1}$	-	5.00 × 10^−5^	$Y_{\frac{G}{Et}}$	g·g^−1^	0.93
$K_{Biomass}^{G}$	g·L^−1^	3	$\delta_{Act}^{S.pH2}$	-	8.1 × 10^−12^	$Y_{\frac{G}{But}}$	g·g^−1^	1.1
$\delta_{Biomass}^{G.pH1}$	-	4.00 × 10^−5^	$K_{Act}^{A}$	g·L^−1^	3	$Y_{\frac{G}{CO_{2}}}$	g·g^−1^	2.2
$\delta_{Biomass}^{G.pH2}$	-	9.00 × 10^−7^	$K_{Act}^{B}$	g·L^−1^	3	$K_{d}$	h^−1^	0.000001
$r_{A}^{max}$	h^−1^	0.9	$K_{IAct}$	g·L^−1^	24	$Y_{\frac{A}{Act}}$	g·g^−1^	0.057
$K_{A}^{G}$	g·L^−1^	0.1	$r_{But}^{G.max}$	h^−1^	0.87	$Y_{\frac{A}{Et}}$	g·g^−1^	0.028
$\delta_{A}^{G.pH1}$	-	5.00 × 10^−7^	$r_{But}^{A.max}$	h^−1^	0.79	$Y_{\frac{A}{But}}$	g·g^−1^	0.3
$\delta_{A}^{G.pH2}$	-	5.00 × 10^−7^	$r_{But}^{B.max}$	h^−1^	0.83	$Y_{\frac{A}{CO_{2}}}$	g·g^−1^	0.4
$K_{IA}$	g·L^−1^	2.8	$K_{But}^{G}$	g·L^−1^	3.5	$Y_{\frac{B}{Act}}$	g·g^−1^	0.28
$r_{B}^{max}$	h^−1^	1.8	$\delta_{But}^{S.pH1}$	-	7.00 × 10^−5^	$Y_{\frac{B}{Et}}$	g·g^−1^	0.15
$K_{IG}^{B}$	g·L^−1^	80	$\delta_{But}^{S.pH2}$	-	8.1 × 10^−12^	$Y_{\frac{B}{But}}$	g·g^−1^	0.33
$K_{B}^{G}$	g·L^−1^	30.2	$K_{But}^{A}$	g·L^−1^	3.1	$Y_{\frac{B}{CO_{2}}}$	g·g^−1^	0.4
$\delta_{B}^{G.pH1}$	-	4.00 × 10^−5^	$K_{But}^{B}$	g·L^−1^	3.3	$\gamma_{KH_{2}PO_{4}}$	-	1 × 10^−10^
$\delta_{B}^{G.pH2}$	-	1.00 × 10^−8^	$K_{IBut}$	g·L^−1^	35	$m_{KH_{2}PO_{4}}$	h^−1^	1 × 10^−8^
$K_{IB}$	g·L^−1^	8	$r_{CO_{2}}^{G.max}$	h^−1^	0.35	$\gamma_{K_{2}HPO_{4}}$	-	1.7 × 10^−1^
$r_{Et}^{G.max}$	h^−1^	0.8	$K_{CO_{2}}^{G}$	g·L^−1^	12.5	$m_{K_{2}HPO_{4}}$	h^−1^	9.9 × 10^−3^
$r_{Et}^{A.max}$	h^−1^	0.54	$\delta_{gas}^{S.pH1}$	-	0.002	pKa	-	6.11
$r_{Et}^{B.max}$	h^−1^	0.57	$\delta_{gas}^{S.pH2}$	-	8.1e-12	$K_{a}^{A}$	-	1.7378 × 10^−5^
$K_{Et}^{G}$	g·L^−1^	4.1	$r_{CO_{2}}^{A.max}$	h^−1^	0.1	$K_{a}^{B}$		1.5136 × 10^−5^
$\delta_{Et}^{S.pH1}$	-	3.00 × 10^−5^	$r_{CO_{2}}^{B.max}$	h^−1^	0.1			
$\delta_{Et}^{S.pH2}$	-	8.1 × 10^−12^	$K_{CO_{2}}^{A}$	g·L^−1^	5			
$K_{Et}^{A}$	g·L^−1^	3.5	$K_{CO_{2}}^{B}$	g·L^−1^	5			
$K_{Et}^{B}$	g·L^−1^	3.8	$r_{H_{2}}^{G.max}$	h^−1^	0.19			
$K_{IEt}$	g·L^−1^	20	$K_{H_{2}}^{G}$	g·L^−1^	2			
$r_{Act}^{G.max}$	h^−1^	0.8	$Y_{\frac{G}{X}}$	g·g^−1^	0.42			
$r_{Act}^{A.max}$	h^−1^	0.6	$Y_{\frac{G}{A}}$	g·g^−1^	0.33			
$r_{Act}^{B.max}$	h^−1^	0.75	$Y_{\frac{G}{B}}$	g·g^−1^	0.33			

**Table 2:** Table represents initial values employed for parameters estimation using experimental data (starting pH 6.4). Table also contains validation of initial values during the simulation to predict the production profiles of other two batch fermentation experiments (starting pH 5.7 and 4.4).

Substrate/Product	Initial Values (g·L^−1^)		
	Starting pH 6.4	Starting pH 5.7	Starting pH 4.4
Glucose	43.981	42.226	45.391
Biomass	0.02	0.02	0.02
Acetic acid	0.002	0.002	0.002
Butyric acid	0.004	0.004	0.004
Ethanol	0	0	0
Acetone	0	0	0
Butanol	0	0	0
Hydrogen	0	0	0
Carbon dioxide	0	0	0
KH_2_PO_4_	0.75	0.75	0.75
K_2_HPO_4_	0.75	0.75	0.75

## Data Availability

The data from this research is available upon request to the corresponding author.

## Nomenclature

$K_{CO_{2}}^{A}$	Acetic acid saturation constant for CO_2_ evolution (g·L^−1^)
$K_{CO_{2}}^{A.pH}$	pH-dependent acetic acid saturation constant for CO_2_ evolution (g·L^−1^)
$K_{CO_{2}}^{B}$	Butyric acid saturation constant for CO_2_ evolution (g·L^−1^)
$K_{CO_{2}}^{B.pH}$	pH-dependent butyric acid saturation constant for CO_2_ evolution (g·L^−1^)
$K_{CO_{2}}^{G}$	Glucose saturation constant for CO_2_ evolution (g·L^−1^)
$K_{CO_{2}}^{G.pH}$	pH-dependent glucose saturation constant for CO_2_ evolution (g·L^−1^)
$K_{H_{2}}^{G}$	Glucose saturation constant for H_2_ evolution (g·L^−1^)
$K_{H_{2}}^{G.pH}$	pH-dependent glucose saturation constant for H_2_ evolution (g·L^−1^)
$K_{A}^{G}$	Glucose saturation constant for acetic acid formation (g·L^−1^)
$K_{A}^{G.pH}$	pH-dependent glucose saturation constant for acetic acid formation (g·L^−1^)
$K_{Act}^{A}$	Acetic acid saturation constant for acetone formation (g·L^−1^)
$K_{Act}^{A.pH}$	pH-dependent acetic acid saturation constant for acetone formation (g·L^−1^)
$K_{Act}^{B}$	Butyric acid saturation constant for acetone formation (g·L^−1^)
$K_{Act}^{B.pH}$	pH-dependent butyric acid saturation constant for acetone formation (g·L^−1^)
$K_{Act}^{G}$	Glucose saturation constant for acetone formation (g·L^−1^)
$K_{Act}^{G.pH}$	pH-dependent glucose saturation constant for acetone formation (g·L^−1^)
$K_{B}^{G}$	Glucose saturation constant for butyric acid formation (g·L^−1^)
$K_{B}^{G.pH}$	pH-dependent glucose saturation constant for butyric acid formation (g·L^−1^)
$K_{Biomass}^{G}$	Glucose saturation constant for biomass synthesis (g·L^−1^)
$K_{Biomass}^{G.pH}$	pH-dependent glucose saturation constant for biomass synthesis (g·L^−1^)
$K_{But}^{A}$	Acetic acid saturation constant for butanol formation (g·L^−1^)
$K_{But}^{A.pH}$	pH-dependent acetic acid saturation constant for butanol formation (g·L^−1^)
$K_{But}^{B}$	Butyric acid saturation constant for butanol formation (g·L^−1^)
$K_{But}^{B.pH}$	pH-dependent butyric acid saturation constant for butanol formation (g·L^−1^)
$K_{But}^{G}$	Glucose saturation constant for butanol formation (g·L^−1^)
$K_{But}^{G.pH}$	pH-dependent glucose saturation constant for butanol formation (g·L^−1^)
$K_{Et}^{A}$	Acetic acid saturation constant for ethanol formation (g·L^−1^)
$K_{Et}^{A.pH}$	pH-dependent acetic acid saturation constant for ethanol formation (g·L^−1^)
$K_{Et}^{B}$	Butyric acid saturation constant for ethanol formation (g·L^−1^)
$K_{Et}^{B.pH}$	pH-dependent butyric acid saturation constant for ethanol formation (g·L^−1^)
$K_{Et}^{G}$	Glucose saturation constant for ethanol formation (g·L^−1^)
$K_{Et}^{G.pH}$	pH-dependent glucose saturation constant for ethanol formation (g·L^−1^)
$K_{IA}$	Product inhibition constant for acetic acid formation (g·L^−1^)
$K_{IAct}$	Product inhibition constant for acetone formation (g·L^−1^)
$K_{IB}$	Product inhibition constant for butyric acid formation (g·L^−1^)
$K_{IBut}$	Product inhibition constant for butanol formation (g·L^−1^)
$K_{IEt}$	Product inhibition constant for ethanol formation (g·L^−1^)
$K_{IG}^{B}$	Substrate inhibition constant for butyric acid formation (g·L^−1^)
$K_{IG}^{X}$	Glucose inhibition constant for biomass synthesis (g·L^−1^)
$K_{a}^{A}$	Dissociation constant for acetic acid
$K_{a}^{B}$	Dissociation constant for butyric acid
$K_{d}$	Endogenous metabolism constant for cell growth (h^−1^)
$X^{max}$	Maximum dry cell weight (g·L^−1^)
$Y_{\frac{A}{CO_{2}}}$	Acetic acid consumption yield coefficient with respect to CO_2_ evolution (g·g^−1^)
$Y_{\frac{A}{Act}}$	Acetic acid consumption yield coefficient with respect to acetone formation (g·g^−1^)
$Y_{\frac{A}{But}}$	Acetic acid consumption yield coefficient with respect to butanol formation (g·g^−1^)
$Y_{\frac{A}{Et}}$	Acetic acid consumption yield coefficient with respect to ethanol formation (g·g^−1^)
$Y_{\frac{B}{CO_{2}}}$	Butyric acid consumption yield coefficient with respect to CO_2_ evolution (g·g^−1^)
$Y_{\frac{B}{Act}}$	Butyric acid consumption yield coefficient with respect to acetone formation (g·g^−1^)
$Y_{\frac{B}{But}}$	Butyric acid consumption yield coefficient with respect to butanol formation (g·g^−1^)
$Y_{\frac{B}{Et}}$	Butyric acid consumption yield coefficient with respect to ethanol formation (g·g^−1^)
$Y_{\frac{G}{CO_{2}}}$	Glucose consumption yield coefficient with respect to CO_2_ evolution (g·g^−1^)
$Y_{\frac{G}{A}}$	Glucose consumption yield coefficient with respect to acetic acid formation (g·g^−1^)
$Y_{\frac{G}{Act}}$	Glucose consumption yield coefficient with respect to acetone formation (g·g^−1^)
$Y_{\frac{G}{B}}$	Glucose consumption yield coefficient with respect to butyric acid formation (g·g^−1^)
$Y_{\frac{G}{But}}$	Glucose consumption yield coefficient with respect to butanol formation (g·g^−1^)
$Y_{\frac{G}{Et}}$	Glucose consumption yield coefficient with respect to ethanol formation (g·g^−1^)
$Y_{\frac{G}{X}}$	Glucose consumption yield coefficient with respect to biomass growth (g·g^−1^)
$m_{K_{2}HPO_{4}}$	maintenance coefficients of K_2_HPO_4_ for biomass (h^−1^)
$m_{KH_{2}PO_{4}}$	maintenance coefficients of KH_2_PO_4_ for biomass (h^−1^)
$r_{CO_{2}}^{A.max}$	Maximum rate of CO_2_ evolution from acetic acid (h^−1^)
$r_{CO_{2}}^{B.max}$	Maximum rate of CO_2_ evolution from butyric acid (h^−1^)
$r_{CO_{2}}^{G.max}$	Maximum rate of CO_2_ evolution from glucose (h^−1^)
$r_{H_{2}}^{G.max}$	Maximum rate of H_2_ evolution from glucose (h^−1^)
$r_{Act}^{A.max}$	Maximum rate of acetone formation for acetic acid (h^−1^)
$r_{Act}^{B.max}$	Maximum rate of acetone formation for butyric acid (h^−1^)
$r_{Act}^{G.max}$	Maximum rate of acetone formation for glucose (h^−1^)
$r_{A}^{max}$	Maximum rate of acetic acid formation (h^−1^)
$r_{B}^{max}$	Maximum rate of butyric acid formation (h^−1^)
$r_{But}^{A.max}$	Maximum rate of butanol formation for acetic acid (h^−1^)
$r_{But}^{B.max}$	Maximum rate of butanol formation for butyric acid (h^−1^)
$r_{But}^{G.max}$	Maximum rate of butanol formation for glucose (h^−1^)
$r_{Et}^{A.max}$	Maximum rate of ethanol formation for acetic acid (h^−1^)
$r_{Et}^{B.max}$	Maximum rate of ethanol formation for butyric acid (h^−1^)
$r_{Et}^{G.max}$	Maximum rate of ethanol formation for glucose (h^−1^)
A	Acetic acid concentration (g·L^−1^)
Act	Acetone concentration (g·L^−1^)
B	Butyric acid concentration (g·L^−1^)
But	Butanol concentration (g·L^−1^)
Et	Ethanol concentration (g·L^−1^)
G	Glucose concentration (g·L^−1^)
X	Dry cell weight (g·L^−1^)
pKa	pKa value for buffer system in media
*Greek letters*	
${µ}^{max}$	Maximum specific growth rate (h^−1^)
$\gamma_{K_{2}HPO_{4}}$	Control coefficients for consumption of K_2_HPO_4_
$\gamma_{KH_{2}PO_{4}}$	Control coefficients for consumption of KH_2_PO_4_
$\alpha_{1}$	Control coefficient for phase 1 (acidogenesis)
$\alpha_{2}$	Control coefficient for phase 2 (solventogenesis)
$\delta_{gas}^{S.pH1}$	Equilibrium constants for key enzyme(s) involved in gases (CO_2_ and H_2_) formation from substrate when its active sites act as a base
$\delta_{gas}^{S.pH2}$	Equilibrium constants for key enzyme(s) involved in gases (CO_2_ and H_2_) formation from acetic acid when its active sites act as an acid
$\delta_{A}^{G.pH1}$	Equilibrium constants for key enzyme(s) involved in acetic acid formation from glucose when its active sites act as a base
$\delta_{A}^{G.pH2}$	Equilibrium constants for key enzyme(s) involved in acetic acid formation from glucose when its active sites act as an acid
$\delta_{Act}^{S.pH1}$	Equilibrium constants for key enzyme(s) involved in acetone formation from substrate when its active sites act as a base
$\delta_{Act}^{S.pH2}$	Equilibrium constants for key enzyme(s) involved acetone formation from substrate when its active sites act as an acid
$\delta_{B}^{G.pH1}$	Equilibrium constants for key enzyme(s) involved in butyric acid formation from glucose when its active sites act as a base
$\delta_{B}^{G.pH2}$	Equilibrium constants for key enzyme(s) involved in butyric acid formation from glucose when its active sites act as an acid
$\delta_{Biomass}^{G.pH1}$	Equilibrium constants for key enzyme(s) involved in biomass formation from glucose when its active sites act as a base
$\delta_{Biomass}^{G.pH2}$	Equilibrium constants for key enzyme(s) involved in biomass formation from glucose when its active sites act as an acid
$\delta_{But}^{S.pH1}$	Equilibrium constants for key enzyme(s) involved in butanol formation from substrate when its active sites act as a base
$\delta_{But}^{S.pH2}$	Equilibrium constants for key enzyme(s) involved in butanol formation from substrate when its active sites act as an acid
$\delta_{Et}^{S.pH1}$	Equilibrium constants for key enzyme(s) involved in ethanol formation from substrate when its active sites act as a base
$\delta_{Et}^{S.pH2}$	Equilibrium constants for key enzyme(s) involved in ethanol formation from substrate when its active sites act as an acid
